# Simultaneous Wireless Power Transfer and Secure Multicasting in Cooperative Decode-and-Forward Relay Networks

**DOI:** 10.3390/s17051128

**Published:** 2017-05-16

**Authors:** Jong-Ho Lee, Illsoo Sohn, Yong-Hwa Kim

**Affiliations:** 1 Department of Electronic Engineering, Gachon University, Seongnam, Gyeonggi 13120, Korea; jongho.lee@gachon.ac.kr; 2 Department of Computer Science and Engineering, Seoul National University of Science and Technology, Seoul 01811, Korea; isohn@seoultech.ac.kr; 3 Department of Electronic Engineering, Myongji University, Yongin, Gyeonggi 17058, Korea

**Keywords:** physical layer security, secure multicasting, wireless power transfer, relay networks

## Abstract

In this paper, we investigate simultaneous wireless power transfer and secure multicasting via cooperative decode-and-forward (DF) relays in the presence of multiple energy receivers and eavesdroppers. Two scenarios are considered under a total power budget: maximizing the minimum harvested energy among the energy receivers under a multicast secrecy rate constraint; and maximizing the multicast secrecy rate under a minimum harvested energy constraint. For both scenarios, we solve the transmit power allocation and relay beamformer design problems by using semidefinite relaxation and bisection technique. We present numerical results to analyze the energy harvesting and secure multicasting performances in cooperative DF relay networks.

## 1. Introduction

As a promising technique for energy-constrained wireless communication systems, simultaneous wireless information and power transfer (SWIPT) techniques have been extensively investigated to provide a cost-effective solution for self-sustainability of energy-limited wireless devices [1,2]. The SWIPT schemes have been utilized in various wireless networks [3,4]. In [3], SWIPT-enabled small cells are deployed in heterogeneous cloud small cell networks and energy harvesting revenues have been considered in interference management problems. In particular, references [5,6,7,8] have focused on security issues in SWIPT sytstems and exploited physical layer security techniques to enable simultaneous secure communication and wireless power transfer. In [5], secure beamforming design for a multi-antenna amplify-and-forward (AF) relay was proposed for SWIPT systems. In multiuser multiple-input single-output (MISO) systems, Ng et al. [6] proposed beamforming design to minimize the total transmit power of the system for simultaneous secure communication and power transfer, whereas [7] maximized secrecy rates satisfying given energy harvesting and overall power constraints. It is noteworthy that a single source-destination pair is considered in [5,6,7,8] for secure communication in SWIPT systems.

In this work, secure broadcasting scenarios [9] are considered in SWIPT systems, where an individual secure message is sent to each destination [8] or a common secure message is sent to multiple destinations (i.e., secure multicasting) [10,11]. In particular, we focus on the secure multicasting scenario with wireless power transfer. A common secure message is sent from a source to its desired destinations, while multiple eavesdroppers also exist to overhear the secure message. In general, a rate at which secure messages can be sent from a source to its intended receiver without being leaked to eavesdroppers is termed an achievable secrecy rate. The maximum achievable secrecy rate is defined as the secrecy capacity. In our scenario, energy receivers also exist in the system to harvest energy from the RF transmission. While a source equipped with multiple antennas is considered in [10,11], we consider that a source equipped with a single antenna performs simultaneous secure multicasting and wireless power transfer with the help of multiple decode-and-forward (DF) single-antenna relays located between the source and the receiver nodes [12]. In this work, multiple relays are designed to operate cooperatively to improve the multicast secrecy rate as well as to enlarge the harvested energy at the energy receivers. It is worth mentioning that the exploitation of cooperative relays for SWIPT systems is also shown in [13,14], but a single source-destination pair is considered for secure communication and amplify-and-forward (AF) relays are utilized.

For simultaneous wireless power transfer and secure multicasting in cooperative DF relay networks, we investigate transmit power allocation and relay beamformer design problems under a total power budget, where the overall power consumed by the source and the relays does not exceed a given limit, in the following two scenarios: (1) maximize the minimum harvested energy achieving a given multicast secrecy rate; and (2) maximize the multicast secrecy rate under a minimum harvested energy constraint. Our contribution in this paper is to show that the optimization problems for both scenarios can be solved by a unified framework consisting of semidefinite relaxation [15] and bisection technique [16].

## 2. System Model

**Figure 1:**
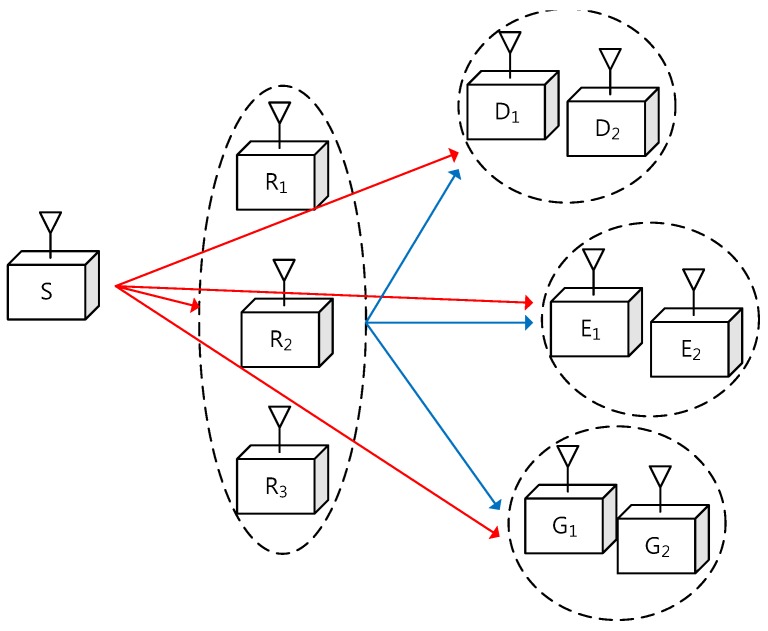
System model for simultaneous wireless power transfer and secure multicasting via cooperative relays (M=3 and I=J=K=2), where *S*, $R_{m}$, $D_{i}$, $E_{j}$, and $G_{k}$ represent the source, the *m*th relay, the *i*th destination, the *j*th eavesdropper, and the *k*th energy receiver, respectively.

As shown in Figure 1, we consider that one source node, *M* trusted relays, *I* destination nodes, *J* eavesdroppers, and *K* energy receivers are deployed in a wireless relay network. All nodes are assumed to have a single antenna and each relay operates in the DF mode. The cooperative relays *R*’s support the source node *S* to send a common secure message to the destination nodes *D*’s. The eavesdroppers *E*’s try to overhear the information message and the energy receivers *G*’s perform energy harvesting from the RF transmission of *S* and *R*’s.

In the first time slot, *S* sends *s* and the other nodes listen. We denote $y_{D_{i}}$, $y_{E_{j}}$, $y_{G_{k}}$, and $y_{R_{m}}$ as the received signals at the *i*th *D*, the *j*th *E*, the *k*th *G*, and the *m*th *R*, respectively, which are given as

(1) $$\begin{array}{cc} y_{D_{i}}= & \sqrt{P_{S}}h_{S,D_{i}}s+z_{D_{i}},\\ y_{E_{j}}= & \sqrt{P_{S}}h_{S,E_{j}}s+z_{E_{j}},\\ y_{G_{k}}= & \sqrt{P_{S}}h_{S,G_{k}}s+z_{G_{k}},\\ y_{R_{m}}= & \sqrt{P_{S}}h_{S,R_{m}}s+z_{R_{m}}, \end{array}$$

where $h_{S,D_{i}}$, $h_{S,E_{j}}$, $h_{S,G_{k}}$, and $h_{S,R_{m}}$ are the complex channel gains from *S* to the *i*th *D*, the *j*th *E*, the *k*th *G*, and the *m*th *R*, respectively, and $P_{S}$ is the transmit power of *S*. Moreover, $z_{D_{i}}$, $z_{E_{j}}$, $z_{G_{k}}$, and $z_{R_{m}}$ are complex additive white Gaussian noises with zero-mean and variance $\sigma^{2}$. In the second time slot, we assume that *R* decode *s* successfully and send the weighted version of the re-encoded symbol. Then, the received signals at *D*’s, *E*’s, and *K*’s can be expressed as

(2) $$\begin{array}{cc} y_{D_{i}}= & h_{R,D_{i}}w_{R}s+z_{D_{i}},\\ y_{E_{j}}= & h_{R,E_{j}}w_{R}s+z_{E_{j}},\\ y_{G_{k}}= & h_{R,G_{k}}w_{R}s+z_{G_{k}}, \end{array}$$

where *M* relay weights are stacked in a M×1 vector $w_{R}$ and $h_{R,D_{i}}$, $h_{R,E_{j}}$, and $h_{R,G_{k}}$ denote 1×M complex channel vectors from *R*’s to the *i*th *D*, the *j*th *E*, and the *k*th *G*, respectively.

Let each *D* and *E* perform maximal ratio combining using the received signals during two time slots. Then, the rates at the *i*th *D* and the *j*th *E* are computed as

(3) $$\begin{array}{cc} R_{D_{i}}= & \frac{1}{2}\log_{2}\left(1+\frac{\alpha_{S,D_{i}}}{\sigma^{2}}P_{S}+\frac{w_{R}^{†}Q_{R,D_{i}}w_{R}}{\sigma^{2}}\right),\\ R_{E_{j}}= & \frac{1}{2}\log_{2}\left(1+\frac{\alpha_{S,E_{j}}}{\sigma^{2}}P_{S}+\frac{w_{R}^{†}Q_{R,E_{j}}w_{R}}{\sigma^{2}}\right), \end{array}$$

where

(4) $$\begin{array}{cc} \alpha_{S,D_{i}}={\left|h_{S,D_{i}}\right|}^{2}, & \alpha_{S,E_{j}}={\left|h_{S,E_{j}}\right|}^{2},\\ Q_{R,D_{i}}=h_{R,D_{i}}^{†}h_{R,D_{i}}, & Q_{R,E_{j}}=h_{R,E_{j}}^{†}h_{R,E_{j}}, \end{array}$$

and ${\left(.\right)}^{†}$ denotes the conjugated transpose. Here, we compute the achievable multicast secrecy rate as [17]

(5) $$R=\max\left\{\min_{i}R_{D_{i}}-\max_{j}R_{E_{j}},0\right\}.$$

The rate at the *m*th *R* is given as

(6) $$R_{R_{m}}=\frac{1}{2}\log_{2}\left(1+\frac{\alpha_{S,R_{m}}}{\sigma^{2}}P_{S}\right),$$

where $\alpha_{S,R_{m}}={\left|h_{S,R_{m}}\right|}^{2}$. Further, the harvested energy at the *k*th *G* is proportional to [5]

(7) $$\Gamma_{k}=\rho \left(P_{S}\alpha_{S,G_{k}}+w_{R}^{†}Q_{R,G_{k}}w_{R}\right),$$

where $\alpha_{S,G_{k}}={\left|h_{S,G_{k}}\right|}^{2}$, $Q_{R,G_{k}}=h_{R,G_{k}}^{†}h_{R,G_{k}}$, and ρ is the energy harvesting efficiency.

In this work, let us assume that global channel state information (CSI) is available. This assumption is valid when we can monitor the transmission of *E*’s since they are also active in the network [18]. In this scenario, *E*’s are low-level users such that they are allowed to access less information than *D*’s. If the global CSI is not available, we may use artificial noise (AN) techniques [19,20], where we consume some portion of the total power budget to transmit artificially generated noises. We expect that our proposed schemes derived in the following can be extended to exploit the AN techniques for simultaneous wireless power transfer and secure multicasting in cooperative DF relay networks.

## 3. Energy Harvesting Maximization with Secure Multicasting Constraints

Our objective in this section is to maximize the minimum harvested energy among *K* energy receivers while guaranteeing that the multicast secrecy rate is greater than or equal to the given threshold (i.e., $R\geq R_{th}$). Because the common information rate for multicasting is determined by the rate of the weakest *S*-*D* link [17,21], we let the rate at each relay be equal to or greater than the minimum rate among *D*’s (i.e., $R_{R_{m}}\geq \min_{i}R_{D_{i}}$ for all *m*) such that each *R* correctly decodes and forwards the common secure message. The optimization problem under a total power budget $P_{T}$ and the DF relaying constraints for secure multicasting is given as

(8) $$\begin{array}{cc} \max_{P_{S},w_{R}} & \min_{k}\Gamma_{k}\\ s.t. & \min_{i}R_{D_{i}}-\max_{j}R_{E_{j}}\geq R_{th},\\ & R_{m}\geq \min_{i}R_{D_{i}},\;\forall m,\\ & P_{S}+w_{R}^{†}w_{R}\leq P_{T},\;P_{S}\geq 0. \end{array}$$

Substituting (3), (6), and (7) into (8), we have

(9) $$\begin{array}{cc} \max_{P_{S},w_{R}} & \min_{k}\rho \left(P_{S}\alpha_{S,G_{k}}+w_{R}^{†}Q_{R,G_{k}}w_{R}\right)\\ s.t. & \frac{\min_{i}\left(\sigma^{2}+\alpha_{S,D_{i}}P_{S}+w_{R}^{†}Q_{R,D_{i}}w_{R}\right)}{\max_{j}\left(\sigma^{2}+\alpha_{S,E_{j}}P_{S}+w_{R}^{†}Q_{R,E_{j}}w_{R}\right)}\geq {\bar{R}}_{th},\\ & \sigma^{2}+\alpha_{S,R}P_{S}\geq \min_{i}\left(\sigma^{2}+\alpha_{S,D_{i}}P_{S}+w_{R}^{†}Q_{R,D_{i}}w_{R}\right),\\ & P_{S}+w_{R}^{†}w_{R}\leq P_{T},\;P_{S}\geq 0, \end{array}$$

where ${\bar{R}}_{th}=2^{2R_{th}}$ and $\alpha_{S,R}=\min_{m}\alpha_{S,R_{m}}$. Let us rewrite (9) as

(10) $$\begin{array}{cc} \max_{P_{S},W_{R},\beta ,\tau_{D},\tau_{E}} & \beta \\ s.t. & P_{S}\alpha_{S,G_{k}}+\mathrm{tr}\left(Q_{R,G_{k}}W_{R}\right)\geq \frac{\beta }{\rho },\;\forall k,\\ & \tau_{D}\geq {\bar{R}}_{th}\tau_{E},\\ & \sigma^{2}+\alpha_{S,D_{i}}P_{S}+\mathrm{tr}\left(Q_{R,D_{i}}W_{R}\right)\geq \tau_{D},\;\forall i,\\ & \sigma^{2}+\alpha_{S,E_{j}}P_{S}+\mathrm{tr}\left(Q_{R,E_{j}}W_{R}\right)\leq \tau_{E},\;\forall j,\\ & \sigma^{2}+\alpha_{S,R}P_{S}\geq \tau_{D},\\ & P_{S}+\mathrm{tr}\left(W_{R}\right)\leq P_{T},\;P_{S}\geq 0,\\ & \mathrm{rank}\left(W_{R}\right)=1,\;W_{R}⪰0,\;\tau_{D}>0,\;\tau_{E}>0, \end{array}$$

where $W_{R}=w_{R}w_{R}^{†}$, tr(.) denotes the trace of a matrix, and $W_{R}⪰0$ represents that $W_{R}$ is a Hermitian positive semidefinite matrix.

In (10), let us ignore the rank constraint using semidefinite relaxation [15]. Then, we use the concept of bisection technique [16] as follows. At first, we set an initial interval [l,u], where the maximum value of β in (10) is assumed to exist. At the midpoint of the given interval $\beta =\frac{l\;+\;u}{2}$, the following feasibility problem is solved by using SeDuMi [22] and Yalmip [23]:

(11) $$\begin{array}{cc} \mathrm{find} & P_{S},W_{R},\tau_{D},\tau_{E}\\ \mathrm{such}\mathrm{that} & P_{S}\alpha_{S,G_{k}}+\mathrm{tr}\left(Q_{R,G_{k}}W_{R}\right)\geq \frac{\beta }{\rho },\;\forall k,\\ & \tau_{D}\geq {\bar{R}}_{th}\tau_{E},\\ & \sigma^{2}+\alpha_{S,D_{i}}P_{S}+\mathrm{tr}\left(Q_{R,D_{i}}W_{R}\right)\geq \tau_{D},\;\forall i,\\ & \sigma^{2}+\alpha_{S,E_{j}}P_{S}+\mathrm{tr}\left(Q_{R,E_{j}}W_{R}\right)\leq \tau_{E},\;\forall j,\\ & \sigma^{2}+\alpha_{S,R}P_{S}\geq \tau_{D},\\ & P_{S}+\mathrm{tr}\left(W_{R}\right)\leq P_{T},\;P_{S}\geq 0,\\ & W_{R}⪰0,\;\tau_{D}>0,\;\tau_{E}>0, \end{array}$$

If (11) is infeasible, we update u=β. If (11) is feasible such that we can obtain the solutions $P_{S}^{⋆}$, $W_{R}^{⋆}$, $\tau_{D}^{⋆}$, and $\tau_{E}^{⋆}$, we check the rank of $W_{R}^{⋆}$. If the rank of $W_{R}^{⋆}$ is one, we update l=β. Otherwise, we exploit the penalty function method (PFM) in [24] with $P_{S}^{⋆}$, $W_{R}^{⋆}$, $\tau_{D}^{⋆}$, and $\tau_{E}^{⋆}$ to confirm the existence of a rank-one solution. The PFM consists of the initialization and optimization steps, which are both an iterative process. In this work, we use $W_{R}^{⋆}$ as a starting point in the initialization step of the PFM. After the iteration of the initialization step is terminated, we may obtain $W_{R}^{\left(0\right)}$ with $\mathrm{rank}\left(W_{R}^{\left(0\right)}\right)\approx 1$. Then, we use $W_{R}^{\left(0\right)}$ as a starting point in the optimization step of the PFM. In the initialization and optimization steps of the PFM, the following semidefinite programming problem is solved at the *l*th iteration:

(12) $$\begin{array}{cc} W_{R}^{\left(l+1\right)}= & {\mathrm{argmin}}_{{\tilde{W}}_{R}}\mathrm{tr}\left({\tilde{W}}_{R}\right)-\lambda_{max}\left(W_{R}^{\left(l\right)}\right)-\mathrm{tr}\left(w_{max}^{\left(l\right)}{\left(w_{max}^{\left(l\right)}\right)}^{†}\left({\tilde{W}}_{R}-W_{R}^{\left(l\right)}\right)\right)\\ s.t. & P_{S}^{⋆}\alpha_{S,G_{k}}+\mathrm{tr}\left(Q_{R,G_{k}}{\tilde{W}}_{R}\right)\geq \frac{\beta }{\rho },\;\forall k,\\ & \sigma^{2}+\alpha_{S,D_{i}}P_{S}^{⋆}+\mathrm{tr}\left(Q_{R,D_{i}}{\tilde{W}}_{R}\right)\geq \tau_{D}^{⋆},\;\forall i,\\ & \sigma^{2}+\alpha_{S,E_{j}}P_{S}^{⋆}+\mathrm{tr}\left(Q_{R,E_{j}}{\tilde{W}}_{R}\right)\leq \tau_{E}^{⋆},\;\forall j,\\ & P_{S}^{⋆}+\mathrm{tr}\left({\tilde{W}}_{R}\right)\leq P_{T},\;{\tilde{W}}_{R}⪰0, \end{array}$$

where $\lambda_{max}\left(W_{R}^{\left(l\right)}\right)$ is the maximum eigenvalue of $W_{R}^{\left(l\right)}$ and $w_{max}^{\left(l\right)}$ denotes the eigenvector of $W_{R}^{\left(l\right)}$ corresponding to the maximum eigenvalue. If we can obtain a rank-one solution using the PFM, we update l=β. Otherwise, we update u=β. Until the width of the updated interval is small enough, the above process continues. The initial interval for the above bisection technique is derived in Appendix A.

## 4. Multicast Secrecy Rate Maximization with Energy Harvesting Constraints

Now, we aim to maximize the achievable multicast secrecy rate [17], while guaranteeing that the minimum harvested energy among *K* energy receivers is greater than or equal to the given threshold (i.e., $\min_{k}\Gamma_{k}\geq \Gamma_{th}$) shown as

(13) $$\begin{array}{cc} \max_{P_{S},w_{R}} & \min_{i}R_{D_{i}}-\max_{j}R_{E_{j}}\\ s.t. & \min_{k}\Gamma_{k}\geq \Gamma_{th},\\ & R_{m}\geq \min_{i}R_{D_{i}},\;\forall m,\\ & P_{S}+w_{R}^{†}w_{R}\leq P_{T},\;P_{S}\geq 0, \end{array}$$

which can be considered as an extended version of [17] for cooperative DF relay networks including the energy harvesting constraints. Substituting (3), (6), and (7) into (13), we have

(14) $$\begin{array}{cc} \max_{P_{S},w_{R}} & \frac{\min_{i}\left(\sigma^{2}\;+\;\alpha_{S,D_{i}}P_{S}\;+\;w_{R}^{†}Q_{R,D_{i}}w_{R}\right)}{\max_{j}\left(\sigma^{2}\;+\;\alpha_{S,E_{j}}P_{S}\;+\;w_{R}^{†}Q_{R,E_{j}}w_{R}\right)}\\ s.t. & \min_{k}\rho \left(P_{S}\alpha_{S,G_{k}}+w_{R}^{†}Q_{R,G_{k}}w_{R}\right)\geq \Gamma_{th},\\ & \sigma^{2}+\alpha_{S,R}P_{S}\geq \min_{i}\left(\sigma^{2}+\alpha_{S,D_{i}}P_{S}+w_{R}^{†}Q_{R,D_{i}}w_{R}\right),\\ & P_{S}+w_{R}^{†}w_{R}\leq P_{T},\;P_{S}\geq 0. \end{array}$$

Let us rewrite (14) as

(15) $$\begin{array}{cc} \max_{P_{S},W_{R},\eta ,\tau_{D},\tau_{E}} & \eta \\ s.t. & \tau_{D}-\eta \tau_{E}\geq 0,\\ & \sigma^{2}+\alpha_{S,D_{i}}P_{S}+\mathrm{tr}\left(Q_{R,D_{i}}W_{R}\right)\geq \tau_{D},\;\forall i,\\ & \sigma^{2}+\alpha_{S,E_{j}}P_{S}+\mathrm{tr}\left(Q_{R,E_{j}}W_{R}\right)\leq \tau_{E},\;\forall j,\\ & \rho \left(P_{S}\alpha_{S,G_{k}}+\mathrm{tr}\left(Q_{R,G_{k}}W_{R}\right)\right)\geq \Gamma_{th},\;\forall k,\\ & \sigma^{2}+\alpha_{S,R}P_{S}\geq \tau_{D},\\ & P_{S}+\mathrm{tr}\left(W_{R}\right)\leq P_{T},\;P_{S}\geq 0,\\ & \mathrm{rank}\left(W_{R}\right)=1,\;W_{R}⪰0,\;\tau_{D}>0,\;\tau_{E}>0. \end{array}$$

As in Section 3, we also exploit the semidefinite relaxation and bisection technique to solve (15). The rank constraint in (15) is ignored and the bisection technique begins with an initial interval [l,u], where the maximum value of η is assumed to exist. At the midpoint of the given interval $\eta =\frac{l\;+\;u}{2}$, the following feasibility problem is solved by using SeDuMi [22] and Yalmip [23]:

(16) $$\begin{array}{cc} \mathrm{find} & P_{S},W_{R},\tau_{D},\tau_{E}\\ \mathrm{such}\mathrm{that} & \tau_{D}-\eta \tau_{E}\geq 0,\\ & \sigma^{2}+\alpha_{S,D_{i}}P_{S}+\mathrm{tr}\left(Q_{R,D_{i}}W_{R}\right)\geq \tau_{D},\;\forall i,\\ & \sigma^{2}+\alpha_{S,E_{j}}P_{S}+\mathrm{tr}\left(Q_{R,E_{j}}W_{R}\right)\leq \tau_{E},\;\forall j,\\ & P_{S}\alpha_{S,G_{k}}+\mathrm{tr}\left(Q_{R,G_{k}}W_{R}\right)\geq \frac{\Gamma_{th}}{\rho },\;\forall k,\\ & \sigma^{2}+\alpha_{S,R}P_{S}\geq \tau_{D},\\ & P_{S}+\mathrm{tr}\left(W_{R}\right)\leq P_{T},\;P_{S}\geq 0,\\ & W_{R}⪰0,\;\tau_{D}>0,\;\tau_{E}>0. \end{array}$$

The bisection process is the same as that described in Section 3. We update u=η when (16) is infeasible. If (16) is feasible, we can obtain $P_{S}^{⋆}$, $W_{R}^{⋆}$, $\tau_{D}^{⋆}$, and $\tau_{E}^{⋆}$. If the rank of $W_{R}^{⋆}$ is one, we update l=η. Otherwise, the PFM is performed to confirm the existence of a rank-one $W_{R}$ for the given $P_{S}^{⋆}$, $\tau_{D}^{⋆}$, and $\tau_{E}^{⋆}$. If we obtain a rank-one solution via the PFM, we update l=η. Otherwise, we update u=η. Until the width of the updated interval is small enough, the above process continues. The initial interval is also derived in Appendix B.

## 5. Numerical Results

**Figure 2:**
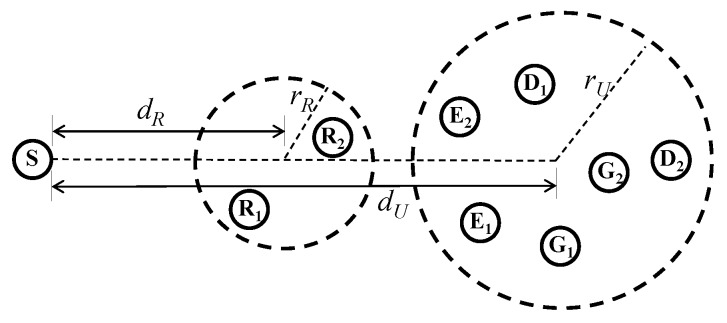
Simulation model.

In this section, we present numerical results to verify the energy harvesting and secure multicasting performance of the proposed schemes. As shown in Figure 2, we assume that *R*’s are located randomly within a circle with a radius of $r_{R}$ and the center of the circle for *R*’s is located $d_{R}$ away from *S*. Further, the radius of a circle, where *D*’s, *E*’s, and *G*’s are located randomly, is $r_{U}$ and the center of this circle is on the line formed by *S* and the center of the circle for *R*’s. The center of the circle for *D*’s, *E*’s, and *G*’s is located $d_{U}$ away from *S*. In our simulation, we set $r_{R}=10$ m, $r_{U}=20$ m, $d_{U}=120$ m, and varied $d_{R}$ for *R*’s to be located between *S* and the receiver nodes *D*’s, *E*’s, and *G*’s as in [12]. We adopt a line-of-sight channel model [12] such that the channel gain between any two nodes is evaluated by $d^{-\frac{c}{2}}e^{j\theta }$, where *d* is the distance between the nodes, c=3.5 denotes the path loss exponent, and θ follows a uniform distribution on [0,2π). For simplicity, we set $P_{T}=40$ dBm and $\sigma^{2}=-30$ dBm. We performed Monte Carlo simulations with $10^{5}$ independent channel realizations and random node locations to obtain the average results.

**Figure 3:**
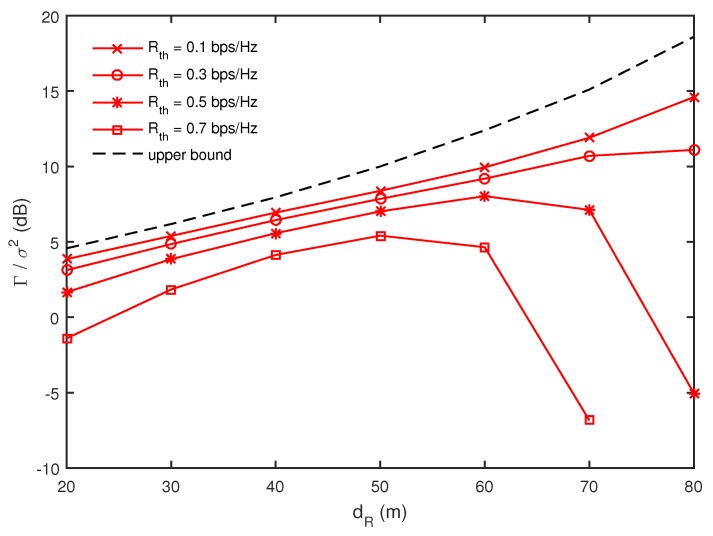
Comparison of minimum harvested energy as a function of $d_{R}$ for different values of $R_{th}$ when M=4 and I=J=K=2.

Let us first consider maximizing the minimum harvested energy under a multicast secrecy rate constraint described in Section 3. In Figure 3, we present $\Gamma /\sigma^{2}$ [7] as a function of $d_{R}$ for different values of $R_{th}$ when M=4 and I=J=K=2, where Γ denotes the average of $\min_{k}\Gamma_{k}$ and the upper bound is the average of the minimum harvested energy without the secure multicast constraints as in Appendix A. Note that only wireless power transfer is performed when there is no secure multicasting constraint, which is equivalent to $R_{th}=0$. In this case, it is beneficial for energy harvesting that *R*’s are located close to *G*’s, because the harvested energy at *G* depends on the received signal strength as shown in (7) and *G* can easily harvest the energy from the RF transmission of the closely located *R*’s. Therefore, the upper bound in Figure 3 increases as *R*’s move closer to the nodes (i.e., $d_{R}$ increases). Now, we focus on a simultaneous wireless power transfer and secure multicasting case with $R_{th}>0$. In this case, *R*’s in the DF mode should decode a secure message from *S* correctly and forward it to *D*’s. Obviously, moving close to *S* is beneficial for *R*’s to decode the secure message from *S* without errors and *R*’s should be located more closely to *S* for larger values of $R_{th}$. Here, we find a tradeoff for the *R*’s location in simultaneous wireless power transfer and secure multicasting scenarios. In our simulation, we do not allow the RF transmission of *S* and *R*’s for a certain channel realization and random location of the nodes, where the given $R_{th}$ cannot be achieved (i.e., the optimization problem in (8) is infeasible for the given $R_{th}$). For smaller values of $R_{th}$, the wireless power transfer is more dominant than the secure multicasting such that the average of the minimum harvested energy Γ still increases as $d_{R}$ increases. However, for larger values of $R_{th}$, Γ is found to decrease drastically for larger values of $d_{R}$. This implies that, when *R*’s are located close to the receiver nodes, it is hard to achieve the given $R_{th}$ for lots of channel realizations and random node locations such that *S* and *R*’s do not perform the RF transmission more frequently and the harvested energy at *G*’s reduces.

**Figure 4:**
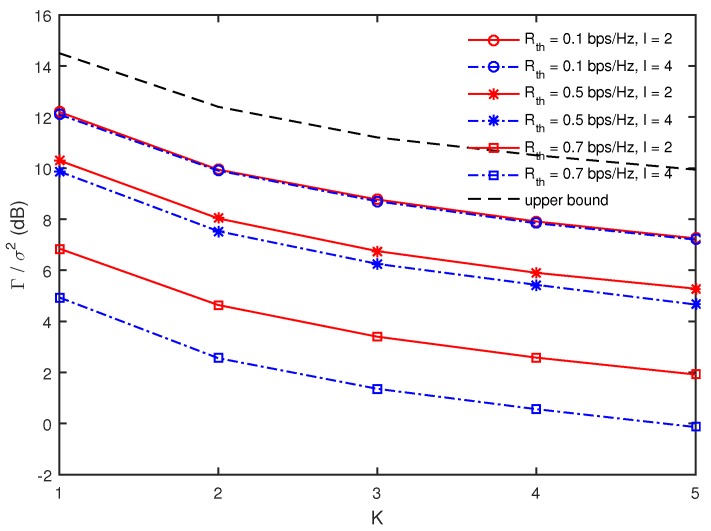
Comparison of minimum harvested energy as a function of *K* for different values of $R_{th}$ and *I* when M=4, J=2, and $d_{R}=60$ m.

Figure 4 presents $\Gamma /\sigma^{2}$ as a function of *K* for different values of $R_{th}$ and *I* when M=4, J=2, and $d_{R}=60$ m. As expected, the minimum harvest energy decreases as *K* increases and also becomes smaller for larger values of $R_{th}$ in all ranges of *K*. However, it is observed that the decrease of the minimum harvested energy according to increasing *K* does not become steeper even though $R_{th}$ is larger. Further, for a given $R_{th}$, the minimum harvested energy with I=2 is greater than that with I=4 in all ranges of *K* and the gap between them becomes larger with an increase of $R_{th}$. Since the multicast secrecy rate depends on the minimum rate among *D*’s as shown in (5), it becomes harder to achieve the given $R_{th}$ as *I* increases. Then, the solution for $P_{S}$ and $w_{R}$ in (8) becomes more oriented to the secure multicasting for achieving $R_{th}$ than the energy harvesting, as *I* becomes larger. This phenomenon becomes severe as $R_{th}$ increases.

**Figure 5:**
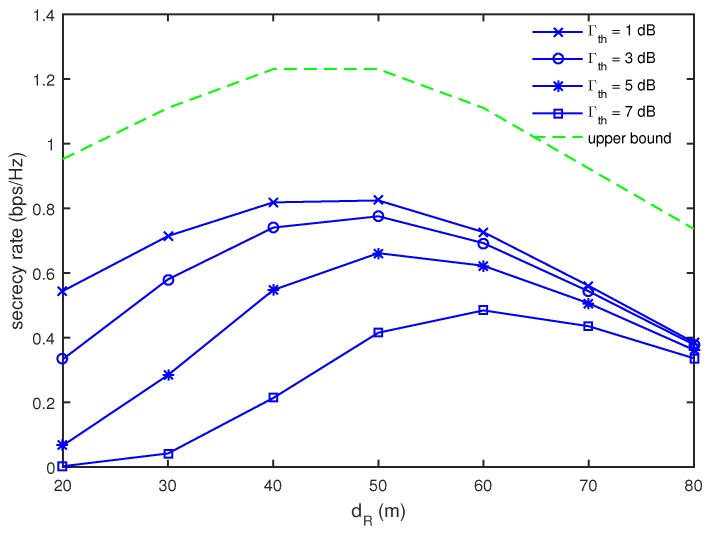
Comparison of multicast secrecy rate as a function of $d_{R}$ for different values of $\Gamma_{th}$ when M=4 and I=J=K=2.

Now, we focus on maximizing multicast secrecy rates under a minimum energy harvesting constraint investigated in Section 4. Figure 5 shows the multicast secrecy rate as a function of $d_{R}$ for different values of $\Gamma_{th}$ when M=4 and I=J=K=2. We evaluated the upper bound in Figure 5 as in Appendix B, which is the multicast rate in the absence of *E*’s without the energy harvesting constraints. Note that *R*’s should decode the signal from *S* correctly as well as forward it to *D*’s thoroughly. In order to decode the signal from *S* correctly, *R*’s should be close to *S*, while it is beneficial for the signal forwarding that *R*’s are located close to *D*. As a compromise, it is optimal for the multicast rate to let *R*’s be in the middle range between *S* and the receiver nodes as shown in the curve for the upper bound in Figure 5. This observation is still valid for simultaneous wireless power transfer and secure multicasting cases. It is seen that the optimal position of *R*’s to provide the maximum multicast secrecy rate is $d_{R}=50$ m until $\Gamma_{th}\leq 5$ dB, while $d_{R}=60$ m is found to be optimal when $\Gamma_{th}=7$ dB. As discussed in Figure 3, it is advantageous for energy harvesting that *R*’s are close to *G*’s. For larger values of $\Gamma_{th}$, the energy harvesting becomes more dominant, such that the optimal position of *R*’s move closer to the receiver nodes. Moreover, the multicast secrecy rate decreases in all ranges of $d_{R}$ as $\Gamma_{th}$ increases. In particular for smaller values of $d_{R}$, the secrecy rate decrease with an increase of $\Gamma_{th}$ is more severe. This implies that, as *R*’s are located farther from *G*’s, it becomes more difficult to satisfy the given energy harvesting constraint.

From the observations in Figure 3 and Figure 5, we found that the location of *R*’s is an important factor to figure out the tradeoff between energy harvesting and secure multicasting in simultaneous wireless power transfer and secure multicasting via cooperative DF relays. In Figure 3, we observed that *R*’s should be close to *G*’s for energy harvesting, while it is advantageous for secure multicasting that *R*’s is located in the middle range of *S* and the receiver nodes as shown in Figure 5. For simultaneous wireless power transfer and secure multicasting in cooperative DF relay networks, we may choose the location of *R*’s to be close to the receiver nodes when the harvested energy is more required than the multicast secrecy rates, while the location of *R*’s may be chosen to be in the middle range between *S* and the receiver nodes when more multicast secrecy rates are needed.

**Figure 6:**
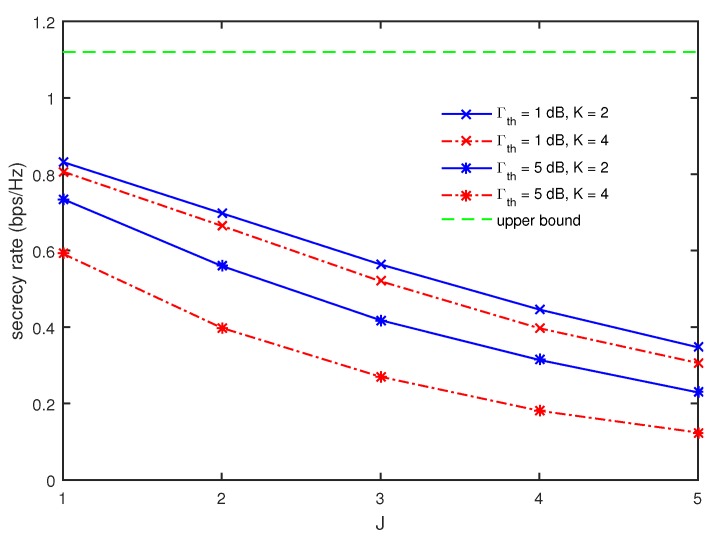
Comparison of multicast secrecy rate as a function of *J* for different values of $\Gamma_{th}$ and *K* when M=4, I=4, and $d_{R}=50$ m.

The multicast secrecy rates are compared in Figure 6 as a function of *J* for different values of $\Gamma_{th}$ and *K* when M=4, I=4, and $d_{R}=50$ m. As expected, the multicast secrecy rate is found to decrease with an increase of *J*. It also becomes smaller for larger values of *K* in all ranges of *J*. Further, it is also observed that the gap between the multicast secrecy rates for K=2 and 4 becomes more significant as $\Gamma_{th}$ becomes larger. Since $\Gamma_{th}$ is the requirement for the minimum harvested energy among *G*’s, it becomes more difficult to achieve $\Gamma_{th}$ as *K* increases. Therefore, with an increase of *K*, the solution for $P_{S}$ and $w_{R}$ in (13) becomes more oriented to the energy harvesting for achieving $\Gamma_{th}$ than the secure multicasting. Further, the increase of $\Gamma_{th}$ brings about the aggravation of this effect.

## 6. Conclusions

In this paper, we considered simultaneous wireless power transfer and secure multicasting in cooperative DF relay networks, where multiple energy receivers and eavesdroppers are deployed. Two different scenarios were investigated such as maximizing the minimum harvested energy under a multicast secrecy rate constraint and maximizing the multicast secrecy rate under a minimum harvested energy constraint. For both scenarios, we showed that the transmit power allocation and relay weight design problems under a total power budget can be solved by a unified framework using semidefinite relaxation and bisection technique. We also presented numerical results to analyze the simultaneous energy harvesting and secure multicasting performances attained by the proposed schemes in cooperative DF relay networks.

## Appendix A

We derive the initial interval for the bisection technique in Section 3. We simply set the lower value of the initial interval as zero. For the upper value of the initial interval, it is reasonable to assume that the harvested energy with the secure multicasting constraint cannot exceed that without the constraint. In this viewpoint, we consider an optimization problem to maximize the minimum harvested energy among *K* energy receivers without the secure multicasting constraints shown as

(A1) $$\begin{array}{cc} \max_{P_{S},W_{R},\beta } & \beta \\ s.t. & P_{S}\alpha_{S,G_{k}}+\mathrm{tr}\left(Q_{R,G_{k}}W_{R}\right)\geq \frac{\beta }{\rho },\;\forall k,\\ & P_{S}+\mathrm{tr}\left(W_{R}\right)\leq P_{T},\;P_{S}\geq 0,\\ & \mathrm{rank}\left(W_{R}\right)=1,\;W_{R}⪰0. \end{array}$$

Ignoring the rank constraint in (A1), we can obtain the solution $P_{S}^{*}$, $W_{R}^{*}$, and $\beta^{*}$ using SeDuMi [22] and Yalmip [23]. Here, we have to check the rank of $W_{R}^{*}$. If the rank is one, $\beta^{*}$ is set to the initial upper value. Otherwise, we use the PFM to confirm the existence of a rank-one solution. As in Section 3, $W_{R}^{*}$ is used as a starting point for the iterative process of the PFM associated with the following semidefinite programming problem:

(A2) $$\begin{array}{cc} W_{R}^{\left(l+1\right)}= & {\mathrm{argmin}}_{{\tilde{W}}_{R}}\mathrm{tr}\left({\tilde{W}}_{R}\right)-\lambda_{max}\left(W_{R}^{\left(l\right)}\right)-\mathrm{tr}\left(w_{max}^{\left(l\right)}{\left(w_{max}^{\left(l\right)}\right)}^{†}\left({\tilde{W}}_{R}-W_{R}^{\left(l\right)}\right)\right)\\ s.t. & P_{S}^{*}\alpha_{S,G_{k}}+\mathrm{tr}\left(Q_{R,G_{k}}{\tilde{W}}_{R}\right)\geq \frac{\beta^{*}}{\rho },\;\forall k,\\ & P_{S}^{*}+\mathrm{tr}\left({\tilde{W}}_{R}\right)\leq P_{T},\;{\tilde{W}}_{R}⪰0. \end{array}$$

If the PFM provides a certificate to confirm that a rank-one solution is feasible, $\beta^{*}$ is set to the initial upper value. Otherwise, let us begin the bisection technique with the initial interval of $\left[0,\beta^{*}\right]$. For the given interval [l,u], $\beta =\frac{l\;+\;u}{2}$ is chosen and the following feasibility problem is solved by using SeDuMi [22] and Yalmip [23]:

(A3) $$\begin{array}{cc} \mathrm{find} & P_{S},W_{R}\\ \mathrm{such}\mathrm{that} & P_{S}\alpha_{S,G_{k}}+\mathrm{tr}\left(Q_{R,G_{k}}W_{R}\right)\geq \frac{\beta }{\rho },\;\forall k,\\ & P_{S}+\mathrm{tr}\left(W_{R}\right)\leq P_{T},\;P_{S}\geq 0,\;W_{R}⪰0. \end{array}$$

If (A3) is infeasible, we update u=β. Otherwise, we check the rank of the solution for $W_{R}$. If the rank is one, we update l=β. Otherwise, we use the PFM to confirm the existence of a rank-one solution. If we can obtain a rank-one solution via the PFM, we update l=β. Otherwise, we update u=β. Until the width of the interval is small enough, the above process continues. After the iteration is terminated, we can obtain our initial upper value.

## Appendix B

For the initial interval of the bisection technique in Section 4, the initial lower value is simply set to one. For the initial upper value, let us consider the multicast rate without the energy harvesting constraints in the absence of the eavesdroppers. Here, we solve the following optimization problem:

(A4) $$\begin{array}{cc} \max_{P_{S},W_{R},\tau_{D}} & \tau_{D}\\ s.t. & \sigma^{2}+\alpha_{S,D_{i}}P_{S}+\mathrm{tr}\left(Q_{R,D_{i}}W_{R}\right)\geq \tau_{D},\;\forall i,\\ & \sigma^{2}+\alpha_{S,R}P_{S}\geq \tau_{D},\\ & P_{S}+\mathrm{tr}\left(W_{R}\right)\leq P_{T},\;P_{S}\geq 0,\\ & \mathrm{rank}\left(W_{R}\right)=1,\;W_{R}⪰0,\;\tau_{D}>0. \end{array}$$

Let us ignore the rank constraint in (A4) to obtain the solution $P_{S}^{*}$, $W_{R}^{*}$, and $\tau_{D}^{*}$ using SeDuMi [22] and Yalmip [23]. Then, we check the rank of $W_{R}^{*}$. If the rank is one, $\tau_{D}^{*}$ is set to the initial upper value. Otherwise, we perform the PFM for the given $P_{S}^{*}$, $W_{R}^{*}$, and $\tau_{D}^{*}$. If the PFM provides a certificate to confirm that a rank-one $W_{R}$ is feasible, we set $\tau_{D}^{*}$ as the initial upper value. Otherwise, we begin the bisection technique with the initial interval of $\left[0,\tau_{D}^{*}\right]$ as in Appendix A. For the given interval [l,u], $\tau_{D}=\frac{l\;+\;u}{2}$ is chosen and the following feasibility problem is solved by using SeDuMi [22] and Yalmip [23]:

(A5) $$\begin{array}{cc} \mathrm{find} & P_{S},W_{R}\\ \mathrm{such}\mathrm{that} & \sigma^{2}+\alpha_{S,D_{i}}P_{S}+\mathrm{tr}\left(Q_{R,D_{i}}W_{R}\right)\geq \tau_{D},\;\forall i,\\ & \sigma^{2}+\alpha_{S,R}P_{S}\geq \tau_{D},\\ & P_{S}+\mathrm{tr}\left(W_{R}\right)\leq P_{T},\;P_{S}\geq 0,\;W_{R}⪰0. \end{array}$$

If (A5) is infeasible, we update $u=\tau_{D}$. Otherwise, we check the rank of the solution for $W_{R}$. If the rank is one, we update $l=\tau_{D}$. Otherwise, we perform the PFM to confirm the existence of a rank-one solution. If we can obtain a rank-one solution via the PFM, we update $l=\tau_{D}$. Otherwise, we update $u=\tau_{D}$. Until the width of the interval is small enough, this process continues. After the iteration is terminated, we can obtain our initial upper value.
